# Immune response in breast cancer brain metastases and their microenvironment: the role of the PD-1/PD-L axis

**DOI:** 10.1186/s13058-016-0702-8

**Published:** 2016-04-27

**Authors:** Renata Duchnowska, Rafał Pęksa, Barbara Radecka, Tomasz Mandat, Tomasz Trojanowski, Bożena Jarosz, Bogumiła Czartoryska-Arłukowicz, Wojciech P. Olszewski, Waldemar Och, Ewa Kalinka-Warzocha, Wojciech Kozłowski, Anna Kowalczyk, Sherene Loi, Wojciech Biernat, Jacek Jassem, for the Polish Brain Metastasis Consortium

**Affiliations:** Department of Oncology, Military Institute of Medicine, Szaserów St 128, 04-141 Warsaw, Poland; Department of Pathology, Medical University of Gdańsk, 7 Dębinki St, 80-211 Gdańsk, Poland; Department of Oncology, Regional Oncology Center, 66a Katowicka St, 45-060 Opole, Poland; Department of Neurosurgery, Oncology Center-Institute, 5 Roentgena St, 02-781 Warsaw, Poland; Department of Neurosurgery, Medical University of Lublin, 1 Al. Racławickie, 20-059 Lublin, Poland; Department of Oncology, Regional Oncology Center, 12 Ogrodowa St, 15-027 Białystok, Poland; Department of Pathology, Oncology Center-Institute, 5 Roentgena St, 02-781 Warsaw, Poland; Department of Neurosurgery, Regional Hospital, 18 Żołnierska St, 10-561 Olsztyn, Poland; Department of Oncology, Regional Oncology Center, 62 Pabianicka St, 93-513 Łódź, Poland; Department of Pathology, Military Institute of Medicine, Szaserów St 128, 04-141 Warsaw, Poland; Department of Oncology and Radiotherapy, Medical University of Gdańsk, 7 Dębinki St, 80-211 Gdańsk, Poland; Division of Cancer Medicine and Research, Peter MacCallum Cancer Centre, Locked Bag 1, A’Beckett Street, East Melbourne, VIC 8006 Australia

**Keywords:** Brain metastases, Breast cancer, PD-1, PD-L, Lymphocytes

## Abstract

**Background:**

A better understanding of immune response in breast cancer brain metastases (BCBM) may prompt new preventive and therapeutic strategies.

**Methods:**

Immunohistochemical expression of stromal tumor-infiltrating lymphocytes (TILs: CD4, CD8, CTLA4), macrophage/microglial cells (CD68), programmed cell death protein 1 receptor (PD-1), programmed cell death protein 1 receptor ligand (PD-L)1, PD-L2 and glial fibrillary acid protein was assessed in 84 BCBM and their microenvironment.

**Results:**

Median survival after BCBM excision was 18.3 months (range 0–99). Median number of CD4+, CD8+ TILs and CD68+ was 49, 69 and 76 per 1 mm^2^, respectively. PD-L1 and PD-L2 expression in BCBM was present in 53 % and 36 % of cases, and was not related to BCBM phenotype. PD-1 expression on TILs correlated positively with CD4+ and CD8+ TILs (*r* = 0.26 and 0.33), and so did CD68+ (*r* = 0.23 and 0.27, respectively). In the multivariate analysis, survival after BCBM excision positively correlated with PD-1 expression on TILs (hazard ratio (HR) = 0.3, *P* = 0.003), CD68+ infiltration (HR = 0.2, *P* < 0.001), brain radiotherapy (HR = 0.1, *P* < 0.001), endocrine therapy (HR = 0.1, *P* < 0.001), and negatively with hormone-receptor-negative/human epidermal growth factor receptor 2 (HER2)-positive phenotype of primary tumor (HR = 2.6, *P* = 0.01), HER2 expression in BCBM (HR = 4.9, *P* = 0.01).

**Conclusions:**

PD-L1 and PD-L2 expression is a common occurrence in BCBM, irrespective of primary tumor and BCBM phenotype. Favorable prognostic impact of PD-1 expression on TILs suggests a beneficial effect of preexisting immunity and implies a potential therapeutic role of immune checkpoint inhibitors in BCBM.

## Background

Breast cancer has not been traditionally considered an immunogenic cancer type. However, there is an increasing body of evidence suggesting that an effective immune response may greatly impact on the clinical behavior of this malignancy. Tumor lymphocyte infiltration is associated with favorable prognosis in early triple-negative and human epidermal growth factor receptor type 2 (HER2)-positive breast cancer phenotypes [1–4] and may influence the response to systemic therapies [3–6]. Information on the association between the immune host response and the colonization of the brain by tumor cells is scarce. The central nervous system (CNS) has long been considered an immunologically privileged site [7]. Actually, CNS is an immune specialized site under a tight regulatory control network linking microglia, astrocytes and lymphocytes [8].

Brain metastases in preclinical and clinical models are characterized by high proliferation, apoptosis, and inflammatory response in the form of surrounding extensive reactive gliosis [9]. It is postulated that the reactive astrocytes reduce apoptosis mediated by the cytotoxic agents by sequestering calcium from the cytoplasm of tumor cells or by secreting metastasis-stimulating chemokines [10]. In the inflammatory and degenerative processes, CNS reactive glial cells actively participate in the restimulation of T cells through the secretion of some chemokines [9, 11, 12]. This increases the influx of regulatory T cell (T_reg_) lymphocytes, resulting in silencing of the immune response.

The programmed cell death protein 1 receptor (PD-1) and its ligands, programmed cell death protein 1 receptor ligand (PD-L)1 and PD-L2, also known as B7-H1 and B7-DC, respectively, play a crucial role in the induction and maintenance of peripheral tolerance, and protect tissues from autoimmune attack [13]. The PD-1/PD-L axis is also a key getaway pathway serving in many cancers as an “immune control” [14, 15]. Several studies suggest that immune response to malignant processes in the brain may be related to the type of cancer [16–19]. Better understanding of the local immune response accompanying brain metastases (BM) may pave the way to the development of novel preventive and therapeutic strategies in breast cancer patients. This retrospective study aimed to assess the correlation between selected parameters of immune response in breast cancer brain metastases (BCBM) and their impact on overall survival.

## Methods

### Study population and data collection

This study was approved by the Institutional Review Board of the coordinating center, the Military Institute of Medicine in Warsaw, Poland. The study group included breast cancer patients who underwent excision of BCBM (Table 1). The patients were diagnosed and treated between 1990 and 2014 in eight oncology centers in Poland. Demographic, clinicopathologic, and clinical follow-up data were extracted from medical records. All data were coded to secure full protection of personal information, therefore, patient consent was not sought.

**Table 1:** Patient characteristics

Variable	*N*	%
Primary tumor and matched brain metastases (*N* = 84)		
Primary tumor histology		
Ductal	70	83
Lobular	7	8
Ductal and lobular	2	2
Other	2	2
Uncertain	3	4
Primary tumor grade		
1	6	7
2	35	42
3	39	46
Unknown	4	5
Primary tumor ERα (IHC)		
Negative	42	50
Positive	42	50
Primary tumor PR (IHC)		
Negative	52	62
Positive	31	37
Unknown	1	1
Primary tumor HER2 (IHC)		
0	21	25
1	18	21
2	9	11
3	36	43
Primary tumor HER2 amplification (FISH; performed in 16 cases)		
No	10	63
Yes	6	37
Primary tumor phenotypes		
HR−/HER2−	21	25
HR+/HER2−	23	27
HR+/HER2+	21	25
HR−/HER2+	19	23
BCBM phenotypes		
HR−/HER2−	24	29
HR+/HER2−	16	19
HR+/HER2+	19	23
HR−/HER2+	24	29
Unknown	1	1
Radiotherapy		
No	24	29
Adjuvant	27	32
Definitive	4	5
Palliative	12	14
Combination thereof	14	17
Unknown	3	6
Chemotherapy		
Induction	52	62
Adjuvant	35	42
For metastatic disease	4	5
Combination thereof	36	43
Unknown	5	6
Endocrine therapy		
No	42	50
Adjuvant	24	29
For metastatic disease	5	6
Combination thereof	11	13
Unknown	2	2
Trastuzumab in HER2+ patients (adjuvant or metastatic setting) before BCBM		
No	19	47
Yes	20	50
Unknown	1	3
Type of first progression		
Regional	5	6
Distant	75	89
Local/regional and distant	3	4
Unknown	1	1
Dominant site of metastatic disease		
Soft tissue	3	4
Bone	4	5
Visceral	76	90
Unknown	1	1
BCBM as first relapse		
No	36	43
Yes	47	56
Number of BCBM		
1	51	61
1–3	20	24
>3	10	12
Unknown	3	4
BCBM sites		
Cerebellum	22	26
Parietal lobe	19	23
Frontal lobe	14	17
Temporal lobe	6	7
Occipital lobe	8	10
Other	2	2
Combination thereof	6	13
Unknown	2	2
Radiotherapy after BCBM excision		
No	15	18
Yes	63	75
Unknown	6	7
Chemotherapy after BCBM excision		
No	38	45
Yes	37	44
Unknown	9	11
Endocrine therapy after BCBM excision in ERα/PR+ primary breast cancer		
No	62	74
Yes	15	18
Unknown	7	8
Trastuzumab after BCBM excision in HER2+ primary breast cancer		
No	32	78
Yes	8	20
Unknown	1	2
Lapatinib after BCBM excision in HER2+ primary breast cancer		
No	14	17
Yes	8	10
Alive at last follow up		
No	75	89
Yes	9	11
Age at breast cancer diagnosis; mean (range) years	49 (28–80)	
Age at BCBM diagnosis; mean (range) years	53 (30–81)	

Percentages for values of patient characteristics may not sum to 100 because of rounding to full numbers. *N* number, *ERα* estrogen receptor alpha, *PR* progesterone receptor, *IHC* immunohistochemical analysis, *FISH* fluorescence in situ hybridization, *HR* hormone receptor, *HER2* human epidermal growth factor receptor 2, *BCBM* breast cancer brain metastases

### Pathologic analysis

The starting material from each patient was an archival formalin-fixed, paraffin-embedded (FFPE) specimen obtained at surgery from the primary breast tumor and BCBM. The pathologic diagnosis was confirmed by a Board-certified pathologist (RP or WB) who reviewed FFPE tissue sections stained with hematoxylin and eosin. A representative paraffin block from each specimen was chosen for immunohistochemical analysis (IHC). In patients with more than one BCBM, only the single most representative lesion was subjected to analysis.

### Immunohistochemical staining

All samples were re-stained and IHC-based expression for estrogen receptor alpha (ERα), progesterone receptor (PR) and HER2 was determined in the central laboratory by two pathologists (RP and WB) who were blinded to the original assessments and to expression in the paired samples. Then, BCBM and the adjacent brain microenvironment were subjected to analysis of stromal tumor infiltrating lymphocytes (TILs) (CD4+, CD8+, CTLA4+), CD68+ cell infiltration, expression of PD-1, PD-L1, PD-L2, and glial fibrillary acid protein (GFAP). The staining was performed according to the manufacturers’ protocols (Table 2). The TILs and CD68+ cells were scored under a light microscope at a magnification of 400 (ocular × 10 with objective of × 40), corresponding to a total area of 1 mm^2^ on full slides. As TILs are predominantly present in stromal parts of BCBM and are a rare occurrence in other BCBM compartments, we have consistently used the term “stromal TILs”. TILs were considered PD-1+ if cytoplasmic staining was found in at least 1 % of cells, irrespective of staining intensity. PD-1 staining was assessed in lymphoid cells, which were identified basing on morphologic features and previously performed staining for/CD4+ and CD8+, with negative results (0) or positive results (1). Due to lack of standardization criteria of PD-L expression positivity and possible intratumoral heterogeneity, PD1-L1 and PD-L2 were assessed in the whole tissue sections using the semiquantitative staining H-score, which accounts for the quantitative and qualitative features of the reactions. The intensity of staining was defined as weak (1), moderate (2), or strong (3). The intensity of the reaction was determined in a percentage of positive cells. The H-score was calculated for each biomarker by the formula:

**Table 2:** Antibodies, dilutions and methods of evaluation

Target	Manufacturer	Catalog number (type of staining)	Dilution	Incubation time	Control tissue	Method of evaluation
PD-1	Novus	NBP1-88104 (cytoplasmic)	1:100	30’ min	LN	SQ
PD-L1	AbD Serotec	AHP2128 (membranous/cytoplasmic)	1:50	40’ min	HSM	SQ
PD-L2	R&D systems	AF1224 (membranous/cytoplasmic)	1:500	30’ min	LN	SQ
CTLA4	Santa Cruz	SC-376016 (cytoplasmic)	1:50	40’ min	LN	SQ
GFAP	Dako	M0761 (cytoplasmic)	RU	30’ min	B	SQ
CD4	Dako	IR649 (membranous)	RU	20’ min	LN	Q
CD8	Dako	IR623 (cytoplasmic and membranous)	RU	20’ min	LN	Q
CD68	Dako	IR609 (cytoplasmic)	RU	20’ min	LN	Q

*RU* ready to use, *SQ* semiquantitative, *Q* quantitative, *HSM* human skeletal muscle, *LN* lymph node, *B* brain, *PD-1* programmed death-1 receptor, *PD-L1* PD-1 ligand 1, *PD-L2* PD-L2 ligand 2, *CTLA4* cytotoxic T cell antigen 4, *GFAP* glial fibrillary acid protein

3 × % Strong cellular staining (cytoplasmic, nuclear and/or membranous) + 2 × % Moderate staining + % Weak staining

**Fig. 1:**
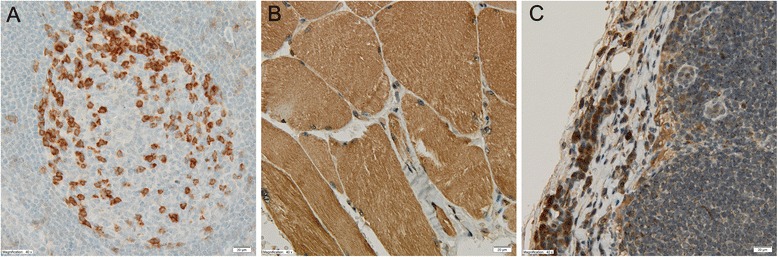
Immunohistochemical positive control (original magnification × 400). **a** Programmed cell death protein 1 receptor (PD-1): lymph node (germinal center in follicle). **b** Programmed cell death protein 1 receptor ligand (PD-L1): skeletal muscle. **c** PD-L2: lymph node (subcapsular sinus)

This gave a range of 0–300. Figure 1 shows positive control staining for PD-1, PD-L1 and PD-L2.

### Statistical analysis

All statistical analyses were performed using STATA software version 11. Statistical significance was defined as *P* < 0.05. We tested correlation between parameters of immune response in BCBM and the brain microenvironment and GFAP, PD-1, PD-L1, PD-L2, TILs (CD4+, CD8+, CTLA4+) and CD68+ cells (listed in Table 3), and assessed their prognostic relevance. This analysis included all available clinical and pathological variables (Table 1). Due to the retrospective and multicenter nature of the study we were unable to include BCBM size and accompanying cerebral edema in the analysis, or the use of steroids and diuretics. Categorical and continuous variables were compared using Pearson’s chi-squared test (*c*^2^), Spearman’s *r* rank test and the Mann–Whitney *U* test. Overall survival (OS) was computed using the Kaplan-Meier method, starting from BCBM excision to the date of death or the last follow up. Univariate and multivariate analyses were performed using the log-rank test, Wilcoxon test, and Cox proportional hazard and logistic regression.

**Table 3:** Assessment of selected parameters of immune response in breast cancer brain metastasis and the brain microenvironment

Variable	Number	%
Reactive astrocytes (glial fibrillary acid protein expression)	83/84	99
No	15	18
Yes	60	71
No neuronal tissue	8	10
Stromal tumor infiltrating lymphocytes^a^ (CD4+)	81/84	96
Median	49	
IQR	23–121	
Stromal tumor infiltrating lymphocytes^a^ (CD8+)	82/84	98
Median	69	
IQR	38–127	
Microglia/macrophages^a^ (CD68+)	77/84	92
Median	76	
IQR	57–104	
PD-1 expression on tumor infiltrating lymphocytes	74/84	88
No	57	68
Yes	17	20
Not determined	10	12
PD-L1 expression on BCBM	78/84	93
H-score; mean (range)	27.1 (0–200)	
PD-L2 expression on BCBM	78/84	93

*IQR* interquartile range, *PD-1* programmed cell death protein 1 receptor, *PD-L1* programmed cell death protein 1 receptor ligand 1, *PD-L2* programmed cell death protein 1 receptor ligand 2, *BCBM* breast cancer brain metastases. ^a^Density was scored at magnification × 400 (ocular × 10 with an objective × 40 high-power field (HPF) per 1 mm^2^

## Results

### Patient characteristics

The study group included 84 breast cancer patients who underwent excision of BM (Table 1). Based on ERα, PR and HER2 expression, four primary tumor phenotypes were identified: hormone-receptor + and HER2– (23 cases), hormone-receptor + and HER2+ (21 cases), hormone-receptor– and HER2– (21 cases), and hormone-receptor– and HER2+ (19 cases). Of these tumors 83 % were invasive ductal carcinomas (no special type); 42 % were grade 2 and 46 % were grade 3: 50 % were ERα– and PR–, 48 % were HER2+ (IHC3+ or *HER2* amplified by fluorescence in situ hybridization (FISH)). All patients underwent radical surgery for the primary tumor; 62 % received neoadjuvant chemotherapy and 42 % adjuvant chemotherapy, 32 % received adjuvant radiotherapy and 19 (50 % of the 38 HER2+ cases) received adjuvant trastuzumab. The first manifestation of progression was distant metastasis in 89 % of patients, with viscera being the most common dominant sites of metastatic disease. Forty-seven patients (56 %) developed BM as the first site of progression, 61 % of whom presented with a single brain lesion at the time of excision. The mean age at BCBM diagnosis was 53 years (range 30–81). The median length of follow up in the entire population was 61.3 months (range 8.7–209 months). The median time to BCBM occurrence from first diagnosis of breast cancer was 41.6 months (range 0.9–152.7). The most common sites of BCBM were the cerebellum and parietal lobe. After BCBM excision, 75 % of patients were administered whole brain radiotherapy, 44 % received chemotherapy and 18 % endocrine therapy. Eight HER2+ patients received trastuzumab or lapatinib. The median OS after BCBM excision was 18.3 months (range 0–99 months).

### Lymphocyte subpopulations, microglia/macrophages and reactive astrocyte infiltration in the brain microenvironment

**Fig. 2:**
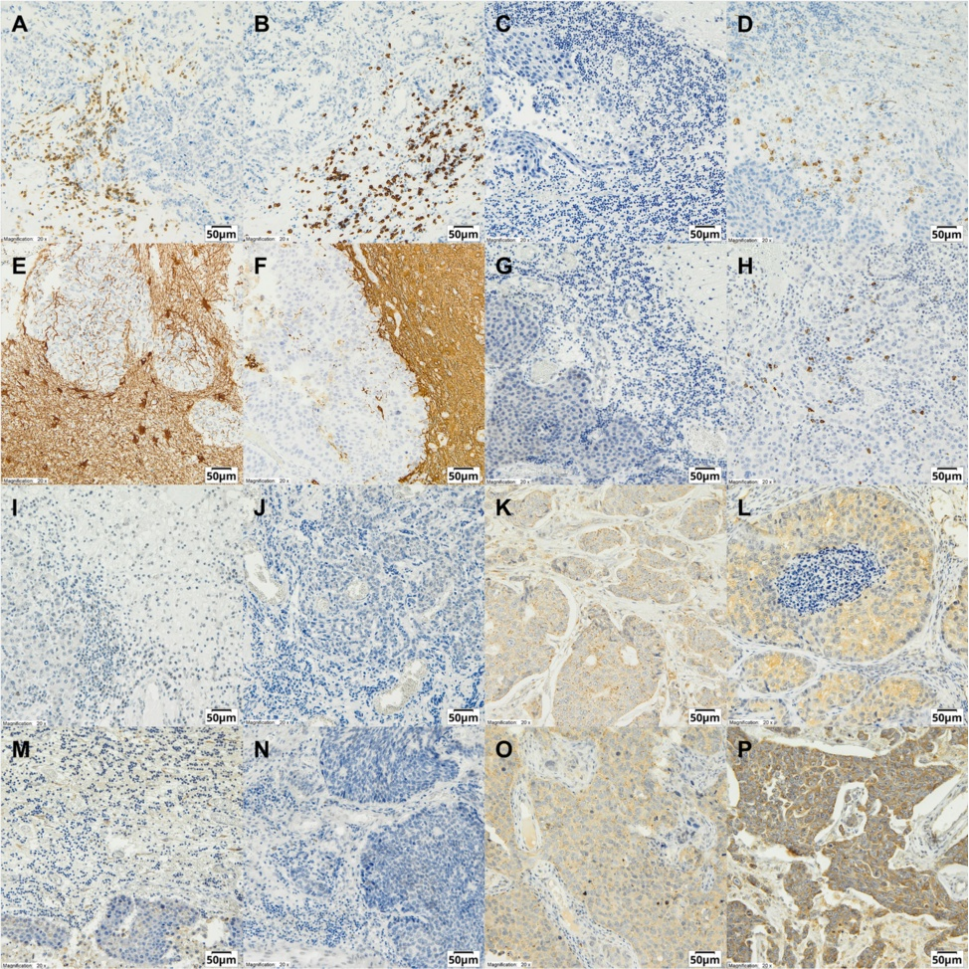
Immunohistochemical analysis (original magnification × 200). **a** CD4+ lymphocytes. **b** CD8+ lymphocytes. **c** CTLA4– lymphocytes. **d** CD68+ cells. **e** Glial fibrillary acid protein (GFAP) weak positive-reactive astrocytes. **f** GFAP moderate positive-reactive astrocytes. **g** Programmed cell death protein 1 receptor (PD-1)-negative expression on stromal tumor infiltrating lymphocytes (TILs) in the brain microenvironment. **h** PD-1-positive expression on TILs in the brain microenvironment. **i** PD-L1 negative expression on TILs in brain microenvironment. **j** Programmed cell death protein 1 receptor ligand (PD-L1)-negative expression in breast cancer brain metastases (BCBM). **k** PD-L1 weak positive expression in BCBM. **l** PD-L1 moderate positive expression in BCBM. **m** PD-L2 negative expression in TILs in the brain microenvironment. **n** PD-L2 negative expression in BCBM. **o** PD-L2 weak positive expression in BCBM. **p** PD-L2 moderate positive expression in BCBM

TIL (CD4+, CD8+) and macrophage/microglia (CD68+) infiltration was determined in 96 %, 98 % and 92 % of cases, respectively (Table 3). TILs were identified in both stromal and epithelial compartments of BCBM, but were generally much more abundant in the stroma (Fig. 2a, b). There was no CTLA4 expression on TILs (Fig. 2c). The median number (per mm^2^) of CD4+ TILs was 49 (interquartile range (IQR) 23–121), of CD8+ TILs was 69 (IQR 38–127), and of CD68+ TILs was 76 (IQR 57–104) (Table 3). CD4+ and CD8+ TILs were positively correlated (*r* = 0.48; *P* < 0.001) and both were positively correlated with CD68+ cells (*r* = 0.23; *P* = 0.043 and *r* = 0.27, *P* = 0.019, respectively) (Table 4). GFAP, a biomarker of reactive astrocytes, was expressed in 71 % of cases (Table 3). There was no correlation between GFAP expression and BCBM phenotype, TILs and CD68+ cell infiltration, or expression of PD-1 and its ligands.

**Table 4:** Spearman’s correlation (*r*) for continuous variables

Variable		CD4	CD8	CD68	PD-L1	PD-L2
CD4	r	–	0.48	0.23	0.12	0.20
	*P*		<0.001	0.043	0.311	0.088
CD8	r	0.48	–	0.27	0.13	0.19
	*P*	<0.001		0.019	0.264	0.100
CD68	r	0.23	0.27	–	0.09	0.19
	*P*	0.043	0.019		0.471	0.104
PD-L1	r	0.12	0.13	0.09	–	0.12
	*P*	0.311	0.264	0.471		0.317
PD-L2	r	0.20	0.19	0.19	0.12	–
	*P*	0.088	0.100	0.104	0.317	
PD-1^a^	r	0.26	0.33	0.06	−0.04	0.13
	*P*	0.028	0.005	0.617	0.742	0.267

*PD-1* programmed cell death protein 1 receptor, *PD-L1* programmed cell death protein 1 receptor ligand. ^a^Categorical variable

### PD-1 expression on TILs and PD-L1, PD-L2 expression in BCBM

**Fig. 3:**
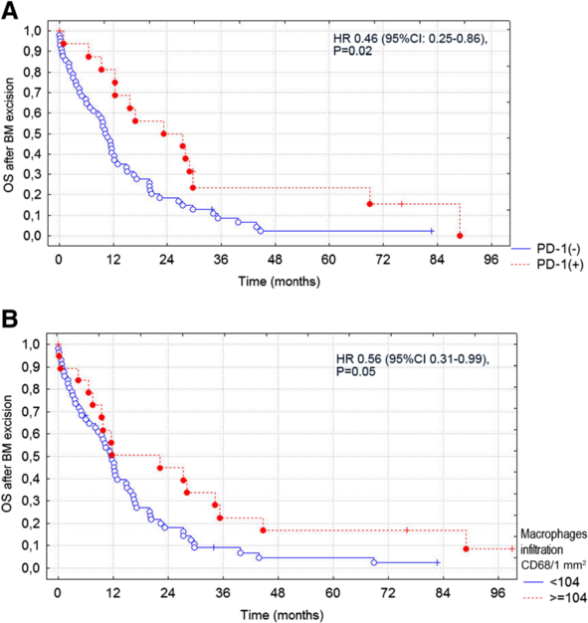
Kaplan-Meier curves for overall survival (*OS*) after excision of breast cancer bone metastases (*BM*). **a** Programmed cell death protein 1 receptor (*PD-1*)-positive vs. PD-1-negative stromal tumor infiltrating lymphocytes (TILs) in the brain microenvironment. **b** high vs. low macrophage/microglia infiltration (CD68+) in the brain microenvironment. *HR* hazard ratio

PD-1 expression on TILs in BCBM was identified in 17 cases (23 %), more frequently in older patients (mean age at brain metastasis diagnosis in PD-1+ and PD-1– groups 59 and 51 years, respectively; *P* = 0.003), and in cases with *HER2*-amplified primary breast cancer. PD-1 expression was correlated positively with both TILs: CD4+ (*r* = 0.26; *P* = 0.028) and CD8+ (*r* = 0.33; *P* = 0.005; Table 4). PD-1+ patients, compared to PD-1– patients had longer OS after BCBM excision (median 27.9 months (range 0.1–88.9) vs. 13.9 months (0.0–82.6), respectively; *P* = 0.02) (Fig. 3a and Table 5). There was no correlation between expression of PD-1 on TILs and PD-1 ligands in BCBM (Table 4). PD-L1 and PD-L2 expression in BCBM was present in 41 (53 %) and 28 (36 %) cases, respectively, and was not related to BCBM phenotype. The mean expression for PD-L1 and PD-L2 (H-score) was 27 and 26, respectively (Table 3).

**Table 5:** Factors impacting overall survival after excision of breast cancer brain metastases (significant in univariate and multivariate analysis)

Variable	Univariate analysis		Multivariate analysis	
	HR (95 % CI)	*P*	HR (95 % CI)	*P*
Primary tumor phenotype HER2-enriched, yes vs. no	3.0 (1.1–9.9)	0.033	2.6 (1.3–5.5)	0.010
Radiotherapy after BCBM, yes vs. no	0.1 (0.0–0.2)	<0.001	0.1 (0.1–0.3)	<0.001
Endocrine therapy after BCBM excision, yes vs. no	0.1 (0.0–0.2)	<0.001	0.1 (0.0–0.3)	<0.001
HER2 IHC expression in BCBM, yes vs. no	6.5 (1.5–27.8)	0.011	4.9 (1.3–19.2)	0.020
PD-1 expression on TILs in brain, yes vs. no	0.5 (0.3–0.9)	0.015	0.3 (0.1–0.7)	0.003
Microglia/macrophages infiltration, ≥104 vs. <104 per mm^2^	0.6 (0.3–1.0)	0.049	0.2 (0.1–0.5)	<0.001

*HR* hazard ratio, *HER2* human epidermal growth factor receptor 2, *BCBM* breast cancer brain metastases, *IHC* immunohistochemical analysis *TILs* stromal tumor-infiltrating lymphocytes, *PD-1* programmed death receptor type 1

### Clinical outcomes

There was no impact of previous systemic adjuvant therapy including trastuzumab on the analyzed immunological parameters in the brain. In HER2+ patients, the administration of trastuzumab before BCBM development did not affect CD4+ (*P* = 0.77) or CD8+ TILs (*P* = 0.17), CD68+ infiltration (*P* = 0.77), or expression of PD-1 (*P* = 0.85), PD-L1 (*P* = 0.86), or PD-L2 (*P* = 0.80). The univariate analysis of survival included all available clinicopathologic variables (the histology, grade and expression of ER, PR and HER2 in the primary tumor, phenotype of the primary tumor and BCBM, treatments administered prior to and after BCBM, type of first progression, dominant metastatic site and location of BCBM) and all studied immune parameters (reactive astrocytes (GFAP), TILs (CD4+, CD8, microglia/macrophages (CD68+), PD-1 expression on TILs, and PD-L1 and PD-L2 expression on BCBM). The multivariate analysis included variables that were significant (*P* < 0.05) in the univariate analysis (Table 5). In this analysis, favorable factors for survival after BCBM excision included PD-1 expression on TILs (hazard ratio (HR) = 0.3 (0.1–0.7); *P* = 0.003) (Fig. 3a) and CD68+ cell infiltration (HR = 0.2 (0.1–0.5); *P* < 0.001) (Fig. 3b), brain radiotherapy (HR = 0.1 (0.1–0.3); *P* < 0.001), and endocrine therapy after the development of BCBM (HR = 0.1 (0.1–0.3) *P* < 0.001; Table 5). Adverse prognostic factors included a hormone receptor–/HER2+ primary tumor phenotype (HR = 2.6 (1.3–5.5); *P* = 0.01) and HER2 expression in BCBM (HR = 4.9 (1.3–19.2); *P* = 0.01).

## Discussion

We have presented a comprehensive analysis of several immune parameters in BCBM. This is also the largest study analyzing the clinical relevance of these parameters. Our data indicate that the PD-1/PD-L axis may play an important role in the local immune response accompanying BCBM. Furthermore, we observed that the infiltration of the brain microenvironment by CD4+ and CD8+ lymphocytes, macrophages/microglia and reactive astrocytes is a common occurrence, and these features are probably independent of BCBM phenotype and previous systemic therapies.

There are two leading hypotheses explaining PD-L1 expression in tumors: the first based on the innative, and the second on the adaptive model [20]. In the innative model, PD-L1 expression is independent of the tumor microenvironment and is influenced by intrinsic cell signaling pathways. The adaptive model assumes that TILs are the key factor driving PD-L1 expression and that immune resistance is exerted by tumor cells in response to endogenous antitumor activity [13, 21–23]. This allows tumor cells to escape immune destruction despite endogenous antitumor immune reactions. Previous studies showed that the PD-1/PD-L axis regulates the induction and maintenance of peripheral tolerance and protects tissues from autoimmune attack (reviewed by Jin et al. [23]). PD-L1 expression in the CNS was identified in glioblastoma and in human brain metastases from melanoma, renal cell carcinoma, lung cancer, colon cancer, and breast cancer, and the PD-1/PD-L1 axis in primary brain lymphomas [16–19]. Here, we demonstrated that PD-L1 and PD-L2 expression is also a common occurrence in BCBM, irrespective of the primary tumor and brain metastasis phenotype.

Recently, PD-L1 expression was found to be more common in primary triple-negative breast cancer [24, 25], but we did not find such a correlation in BCBM. However, PD-L1 expression is a dynamic process in normal conditions and is influenced by cytokines, such as interferon (INF)-γ [26]. In turn, PD-L1 expression in tumor cells may be influenced by systemic therapy. Moreover, biopsy timinig (at diagnosis vs. at progression) to determine PD-L1 expression may be critical in patient selection for immune checkpoint inhibitors or other experimental therapies [27]. Hitherto, there are no data on the comparison of PD-L expression in primary breast cancer and in the corresponding BCBM. In a recent study by Berghoff et al. [28] there was no correlation between PD-L1 expression in brain metastases from various solid tumors and TIL density, and the authors also postulated that the density of CD3+, CD8+ and CD45RO+ TILs, and the calculated immunoscore, are positively correlated with survival. In the recent study by Harter et al. [19], which included several tumor types, there was no significant prognostic impact of TIL expression in brain metastases in the entire population, and there was a strong trend towards better survival in brain metastases from melanoma with high levels of PD-L1 [19].

The biological role of PD-L2 is less well-understood. Recent studies showed that PD-L2 can be induced on antigen-presenting cells, such as macrophages, dendritic cells, T cells and a wide variety of non-immune cells, depending on the microenvironmental stimuli [29]. Some studies suggest an adverse prognostic impact of PD-L expression, whereas others, including ours, did not find such a relationship, or even showed the opposite [18, 19, 30, 31]. These differences may likely be due to different methods used for the detection of ligand expression and the lack of standardized criteria for assessment of PD-L expression.

PD-1 is an inhibitory co-receptor expressed on activated and exhausted T cells [13–15, 32, 33]. We demonstrated that PD-1 expression on TILs in BCBM is independently associated with increased OS. However, our study included patients with limited numbers of BCBM eligible for resection and with good performance status, and most had controlled extracranial disease. Hence it is unknown whether this observation applies to all patients with BCBM. Although PD-1 expression correlated with CD4+ and CD8+ TILs, increased OS was not directly related to the mere presence of TILs, an observation suggesting the importance of preexisting active immunity. Interestingly, in the abovementioned study by Harter et al. [19], PD-1+ lymphocytes and the ratio between PD-1 and CD8+ cells were higher in smaller than in larger metastases. This finding may indicate that in smaller metastases the lymphocytic immune response is activated but functionally impaired. It is also possible that T cells may control the tumor size transiently before becoming exhausted.

Data on the prognostic value of PD-1 expression on TILs in various malignancies are scarce and inconsistent. In primary renal cell carcinoma, on univariate analysis PD-1 expression on mononuclear immune cell infiltrates was found to increase the risk of cancer-specific death and overall mortality [34]. However, in this study PD-1 was associated with more advanced disease, the presence of coagulation, tumor necrosis, and sarcomatoid differentiation. Hence, this feature may be associated with more aggressive disease characteristics rather than be an adverse prognostic factor per se. Similarly, in operable breast cancer, PD-1+ immune cell infiltration in the primary tumor is reported to correlate with shorter survival [35]. In contrast to these reports, in a series of recent studies the PD-1/PD-L1 axis had a favorable effect, supporting the role of preexisting antitumor immunity [5, 36, 37]. Notably, all these studies relate to primary tumors, whereas we included BCBM, in which immune mechanisms may be substantially different due to the immune privilege of the CNS [7, 37]. Nonetheless, evidence of a favorable prognostic role of PD-1 expression on TILs in BCBM should be considered cautiously and warrants confirmation.

We did not observe a relationship between expression of PD-1 on TILs, and PD-Ls expression in BCBM, and neither did we find major differences across breast cancer phenotypes, except for more common PD-1 expression in *HER2*-amplified primary tumors. According to the adaptive resistance hypothesis, cancer cells can upregulate the expression of PD-L1 after encountering T cells, mostly via IFN-γ. However, there are data suggesting that cancer cells also express PD-L1 by an intrinsic, INF-γ independent mechanism [38, 39]. Further, some genetic abnormalities, such as a loss of phosphatase and tensin homolog in glioma or triple-negative breast cancer, and epidermal growth factor receptor mutations in lung cancer, can directly upregulate PD-L1 on cancer cells [24, 40, 41]. On the other hand, it has been speculated that the local CNS microenviroment may in some way suppress the INF-γ mediated response, thus, paradoxically decreasing brain tissue damage [37]. Interestingly, only an undetermined fraction of lymphocyte infiltration dies through the interaction with the PD-1/PD-L axis. Additionally, there are non-PD-1 costimulatory receptors for PD-L, which are responsible for the enhanced effector function of PD-L-expressing tumor cells [42, 43].

In this series, besides PD-1 expression, macrophages/microglia infiltration was also found to be associated with significantly longer survival after the excision of BCBM. The macrophages/microglia play a key role in the development of innate and adaptive immune response in the brain [44]. These cells are perceived as a main source of proinflammatory cytokines and more as antigen-presenting cells, and actively participate in the T cell restimulation [8, 9, 44]. The limitation of our study was identifying macrophages/microglia exclusively by CD68 staining, as other markers (such as CD14, CD11b, and/or MHC-II) might have likely provided more data on the prognostic role of these cells.

Some preclinical studies suggest a potential role for immune checkpoint inhibitors in mammary tumors, particularly HER2+ phenotypes. Combining trastuzumab with inhibitors of negative T cell regulation, such as anti-PD-1, anti-PD-L1 or anti-CTLA4 antibodies, may increase antitumor efficacy [45, 46]. In HER2+ patients receiving trastuzumab, PD-1 inhibition stimulates CD8+ cells producing INF-γ, and may increase the therapeutic effect of this antibody [46]. However, in our study trastuzumab administered before the development of BCBM did not affect the expression of TILs, CD68+ cell infiltration, or PD-1 and its ligands in BCBM. The brain microenvironment may promote HER2 expression via secretion of specific cytokines, such as neuregulin [47]. We recently demonstrated that expression of quantitative HER2 and p95 - its truncated, constitutively active form - is significantly increased in BCBM compared to primary breast cancers [48]. In that study, p95 expression in brain metastases also correlated with poorer clinical outcome.

Currently, PD-1 inhibitors, such as pembrolizumab and nivolumab, are a subject of clinical investigation in non-small cell lung cancer and melanoma with brain metastases (NCT02085070, NCT02320058), whereas no data are available for anti-PD therapies in BCBM. Hence, there is a rationale for investigation into boosting the host antitumor immune response by inhibiting the inhibitors (via increasing lymphocyte influx to the brain or inhibiting PD-L expression in tumor cells) also in BCBM. Pembrolizumab has shown promising effects and a good safety profile in PD-L1-positive advanced triple-negative breast cancer (KEYNOTE-012 study; NCT01848834) and in heavily pretreated ER+/HER2– breast cancer (KEYNOTE-028 study; NCT02054806) [49, 50]. PD-L1 inhibitors, atezolizumab (MPDL3280A) and avelumab (MSB0010718C) appear to be particularly active in triple-negative breast cancer [51, 52]. Several ongoing clinical trials are investigating other immune checkpoint inhibitors in both in locally advanced and/or metastatic breast cancer and in the adjuvant setting (reviewed in Chawla et al. [53]).

## Conclusions

We demonstrated an important role for an activated preexisting immune response in a relatively large group of patients with BCBM. However, we are aware of some limitations of this study, including its retrospective nature, small number of cases in particular subsets, and the lack of assay standardization in terms of sampling and other technical issues. Thus, our findings warrant confirmation in further investigations.

## Abbreviations

BCBM: breast cancer brain metastases
BM: brain metastases
CNS: central nervous system
ERα: estrogen receptor alpha
FFPE: formalin-fixed, paraffin-embedded
GFAP: glial fibrillary acid protein
HER2: human epidermal growth factor receptor 2
IHC: immunohistochemistry
INF: interferon
IQR: interquartile range
OS: overall survival
PD-1: programmed cell death protein 1 receptor
PD-L: programmed cell death protein 1 receptor ligand
PR: progesterone receptor
TILs: stromal tumor infiltrating lymphocytes
